# Autoimmune Diseases in the Bioinformatics Paradigm

**DOI:** 10.1016/j.gpb.2015.09.003

**Published:** 2015-10-01

**Authors:** Quan-Zhen Li, Edward K. Wakeland

**Affiliations:** Department of Immunology and Internal Medicine, University of Texas Southwestern Medical Center, Dallas, TX 75235, USA

Autoimmune diseases (AIDs) consist of a group of physiological disorders with highly diversified pathogenesis and clinical manifestations [1], which affect more than 5% of the population worldwide [2]. So far, the etiology of the AIDs is still poorly understood, whereas it is generally believed that autoimmune disorder results from a complex interaction of genetic and epigenetic variations, as well as triggering environmental factors [3]. Because of the varied phenotypes in different individuals of one AID and sometimes shared manifestations among patients with different AIDs, precise diagnoses, prognosis, and effective treatment are hard to achieve. In the last decade, with the explosion of multi-omics technologies, such as genomics, transcriptomics, epigenetics, proteomics, and metabolomics, the new high-throughput approaches have been widely applied in the studies of AIDs and led to the discovery of various biomarkers with the potential to facilitate the precise medicine in a big data paradigm.

In this special issue, we aimed to give an overview on the most recent progress of biomarker studies on some of the important autoimmune-related diseases, such as systemic lupus erythematosus (SLE), psoriasis, systemic sclerosis (SSc), and primary Sjogren’s syndrome (pSS). Dr. Tianfu Wu’s group [4] provided a comprehensive review on the diagnostic biomarkers of psoriasis, a chronic skin disease that affects around more than 7.2 million people solely within the US in 2013 [5]. The biomarker discovery is especially essential for this disease due to the heterogeneous therapeutic reactions among individuals and the lack of cure, thus precise medicine might be achieved through understanding of individuals’ immune responses. The efforts of identifying suitable psoriasis biomarkers have been continuously made using different methods, including conventional analyses and omics approaches (genomics, transcriptomics, proteomics, and metabolomics), with the fast-developing technologies as reviewed in this paper.

The prevalence of pSS is hard to be estimated due to the inconsistent classification criteria of the disease. It also varies largely among surveys in different age groups, ethnicities, and geographical regions with an obvious bias in female population [6]. Because of the phenotypic variances, diagnostic biomarkers, especially non-invasive biomarkers, are valuable for the early definitive diagnosis. Dr. Song-Guo Zheng’s group [7] reviewed the recent progress of recruiting interferon type I and Fms-like tyrosine kinase 3 ligand as the pSS biomarkers, as well as the potential to find microRNA biomarkers using genomics approaches.

SSc is a chronic multisystem AID with an estimated prevalence in the US to be around 240 cases/million adults [8]. With the recent blooming of the microRNA studies, the epigenetic effects on SSc have been extensively studied in the transforming growth factor-β (TGF-β) pathway. Dr. Xiaoxiao Zuo’s group [9] conducted an in-depth review specifically focused on the epigenetic regulation on SSc and pointed out the promising direction for new SSc biomarkers.

SLE is the prototypic AID predominately affecting woman with the production of various autoantibodies as one of the major clinical manifestations. Proteomic microarray, as a powerful high throughput autoantibody screening approach, has been extensively utilized for identification of autoantibodies associated with pathogenesis of SLE. A review from Dr. Quan-Zhen Li’s group [10] discussed the most recent progresses on the application of autoantigen microarrays for profiling of autoantibodies in SLE and their potential application as biomarkers for early diagnosis and therapeutic intervention.

The review from Dr. Haitao Niu’s group [11], on the other hand, focused on using next-generation sequencing (NGS) technology to identify biomarkers for several AIDs, such as SLE, SSc, and rheumatoid arthritis (RA). Other than the review articles on various AIDs, Dr. Yigang Tong [12] also highlighted a recently-published study on the altered microbiomes in RA patients, which could potentially serve as a biomarker for disease diagnosis and treatment response [13]. Two original articles are included to enlarge the scope of this special issue: Dr. Bruce Budowle [14] introduced their work on short tandem repeat of Y-chromosome using the massively parallel sequencing and Dr. Changqing Zeng’s group [15] reported the genome-wide characteristics of esophageal squamous cell carcinoma.

The multiple omics technologies have greatly facilitated the discovery of biomarkers and improved our knowledge of the etiopathology of AIDs. In the meantime, the advances in high-throughput screening technologies offered excellent opportunities to identify new biomarkers potentially useful for early diagnosis, prognosis, and even prediction of therapeutic responses. Given the high complexities of the immune system and diversification of AIDs, the exploration of novel biomarkers and their underlying molecular mechanism will continue to be hot topics in the study of AIDs. Putting these articles together in this special issue might be helpful to reveal the intercommunication of the AIDs in the new bioinformatics and big data picture and may shed light on other less-studied AIDs or the discovery of universal biomarkers. In addition, novel biomarkers, especially the non-invasive biomarkers, are greatly demanded. Methodologically, application of the state-of-art novel technologies, such as NGS, as well as high-throughput proteomics and metabolomics approaches, in combination with various data analysis platforms, would greatly facilitate the discovery of multiple disease biomarkers and assessment of genes, proteins, metabolites, and network analysis of complex pathways implicated in the specific AID conditions. Comprehensive understanding of the relationship between the biomarkers and AIDs will not only provide valuable tools for disease prediction, early diagnosis, and prognosis, but also shed light on the identification of novel therapeutic target for treatment of the diseases.

## Competing interests

The authors have declared no competing interests.

## References

[1] Teixeira P.C., Ferber P., Vuilleumier N., Cutler P. (2015). Biomarkers for cardiovascular risk assessment in autoimmune diseases. Proteomics Clin Appl.

[2] Cooper G.S., Bynum M.L., Somers E.C. (2009). Recent insights in the epidemiology of autoimmune diseases: improved prevalence estimates and understanding of clustering of diseases. J Autoimmun.

[3] Ramos P.S., Shedlock A.M., Langefeld C.D. (2015). Genetics of autoimmune diseases: insights from population genetics. J Hum Genet.

[4] Jiang S., Hinchliffe T.E., Wu T. (2015). Biomarkers of an autoimmune skin disease — psoriasis. Genomics Proteomics Bioinformatics.

[5] Rachakonda T.D., Schupp C.W., Armstrong A.W. (2014). Psoriasis prevalence among adults in the United States. J Am Acad Dermatol.

[6] Patel R., Shahane A. (2014). The epidemiology of Sjögren’s syndrome. Clin Epidemiol.

[7] Chen W., Cao H., Lin J., Olsen N., Zheng S.G. (2015). Biomarkers for primary Sjögren’s syndrome. Genomics Proteomics Bioinformatics.

[8] Korman B.D., Criswell L.A. (2015). Recent advances in the genetics of systemic sclerosis: toward biological and clinical significance. Curr Rheumatol Rep.

[9] Li Y., Huang J., Guo M., Zuo X. (2015). MicroRNAs regulating signaling pathways: potential biomarkers in systemic sclerosis. Genomics Proteomics Bioinformatics.

[10] Zhu H., Luo H., Yan M., Zuo X., Li Q.Z. (2015). Autoantigen microarray for high-throughput autoantibody profiling in systemic lupus erythematosus. Genomics Proteomics Bioinformatics.

[11] Ma Y., Shi N., Li M., Chen F., Niu H. (2015). Applications of next-generation sequencing in systemic autoimmune diseases. Genomics Proteomics Bioinformatics.

[12] Tong Y. (2015). Metagenome-wide association studies potentiate precision medicine for rheumatoid arthritis. Genomics Proteomics Bioinformatics.

[13] Zhang X., Zhang D., Jia H., Feng Q., Wang D., Liang D. (2015). The oral and gut microbiomes are perturbed in rheumatoid arthritis and partly normalized after treatment. Nat Med.

[14] Warshauer D.H., Churchill J.D., Novroski N., King J.L., Budowle B. (2015). Novel Y-chromosome short tandem repeat variants detected through the use of massively parallel sequencing. Genomics Proteomics Bioinformatics.

[15] Wang Q., Bai J., Abliz A., Liu Y., Gong K., Li J. (2015). An old story retold: loss of G1 control defines a distinct genomic subtype of esophageal squamous cell carcinoma. Genomics Proteomics Bioinformatics.

